# Mindful Self-Compassion Smartphone Intervention for Worker Mental Health in Japan: Protocol for a Randomized Controlled Trial

**DOI:** 10.2196/53541

**Published:** 2024-07-15

**Authors:** Takumu Kurosawa, Koichiro Adachi, Ryu Takizawa

**Affiliations:** 1 Department of Clinical Psychology Graduate School of Education The University of Tokyo Tokyo Japan; 2 MRC Social, Genetic and Developmental Psychiatry Centre Institute of Psychiatry, Psychology and Neuroscience King’s College London London United Kingdom

**Keywords:** self-compassion, mindfulness, smartphone apps, preventive intervention, mental health, work performance, smartphone intervention, workers, psychological support, mindfulness meditation, meditation, work-related outcomes, mobile phone

## Abstract

**Background:**

Mental health problems among workers cause enormous losses to companies in Japan. However, workers have been considered to have limited access to psychological support because of time constraints, which makes it difficult for them to engage in face-to-face psychological support interventions.

**Objective:**

This study aimed to present an intervention protocol that describes a randomized controlled trial to examine whether brief guided mindfulness meditation (MM) or self-compassion meditation (SCM) provided by a smartphone app is effective for mental health and work-related outcomes among workers.

**Methods:**

This is an open-label, 3-arm randomized controlled trial. The participants will be recruited through an open call on relevant websites with the following inclusion criteria: (1) employees who are working more than 20 hours per week, (2) between the ages of 18 and 54 years, (3) not on a leave of absence, (4) not business owners or students, and (5) not currently diagnosed with a mental disorder and have a Kessler Psychological Distress Scale-6 score below 13 points. We will include 200 participants and randomly assign them to an SCM course (n=67), an MM course (n=67), and a waitlist group (n=66). The intervention groups (SCM and MM) will be instructed to engage in daily guided self-help, self-compassion, and MMs lasting 6-12 minutes per day over 4 weeks. Primary outcomes will include psychological distress and job performance, and secondary outcomes will include somatic symptoms, cognitive flexibility, self-esteem, self-compassion, perceived stress, well-being, emotion regulation, work engagement, anger, psychological safety, and creativity. All procedures were approved by the ethics committee of the University of Tokyo (22-326). All participants will be informed of the study via the websites, and written informed consent will be collected via web-based forms.

**Results:**

The recruitment of participants began in December 2022, and the intervention began in January 2023. As of September 2023, a total of 375 participants have been enrolled. The intervention and data collection were completed in late October 2023.

**Conclusions:**

This study will contribute to the development of effective self-care intervention content that will improve mental health, work performance, and related outcomes and promote mindful and self-compassionate attitudes when faced with distress.

**Trial Registration:**

University Hospital Medical Information Network Clinical Trials Registry UMIN000049466; https://tinyurl.com/23x8m8nf

**International Registered Report Identifier (IRRID):**

DERR1-10.2196/53541

## Introduction

### Background and Rationale

Worker health issues significantly influence corporate management due to productivity losses and worsening employee retention. This has led to a social problem in Japan, where the working-age population is decreasing [1]. Mental health problems such as depression, anxiety, and emotional disorders have been reported to cause personal wage loss [2]. All levels of depression are associated with impaired work productivity, and even mild levels of depression have been reported to decrease work performance [3]. The World Health Organization [4] defines mental health as “a state of well-being in which the individual realizes his or her own abilities, can cope with the normal stresses of life, can work productively and fruitfully, and is able to make a contribution to his or her community.” This indicates that being productive is also an essential element of personal well-being. Thus, preventive support for mental health problems is beneficial not only for workers’ mental health but also for their well-being and work performance.

### Mindfulness and Self-Compassion for Workers

Mindfulness and self-compassion have been of particular interest in providing psychological support to workers because of their potential positive effects on work-related outcomes [5]. Mindfulness is defined as “the awareness that emerges through paying attention on purpose, in the present moment, and nonjudgmentally to the unfolding of experience moment by moment” [6]. Mindfulness is also a widespread self-care practice among workers in the United States, with more than 13% reporting that they practice mindfulness [7]. Evidence from previous studies has confirmed that mindfulness interventions have beneficial effects on mental and physical health, including anxiety, depression, chronic pain, and immunity [8,9]. A growing body of evidence demonstrates the effectiveness of mindfulness-based interventions for workers in improving positive indicators, such as work engagement, personal accomplishment, good sleep quality, and relaxation, and reducing negative indicators, such as emotional exhaustion, occupational stress, and anxiety [10,11]. Some researchers have shown that mindfulness training improves key workplace outcomes, such as performance and relationships [12,13]. Mindfulness affects attention and, through attention, influences major domains of human functioning, including cognition, emotion, behavior, and physiology, resulting in improved workplace outcomes such as performance and creativity [13].

Compassion is another area of growing interest in psychotherapy research [14]. Kirby [15] identified at least 8 types of compassion-based therapies that cultivate compassion (eg, compassion-focused therapy, mindful self-compassion, and compassion and loving-kindness meditation). Self-compassion refers to kindness toward oneself in compassion. Neff [16] defined self-compassion as comprising three main components: (1) self-kindness, (2) common humanity, and (3) mindfulness. These elements are described as follows: (1) self-kindness: being kind and understanding toward oneself in instances of pain or failure rather than being harshly self-critical, (2) common humanity: perceiving one’s experiences as part of the larger human experience rather than seeing them as separating and isolating, and (3) mindfulness: holding painful thoughts and feelings in balanced awareness rather than overidentifying with them [16]. Meta-analyses of compassion-based interventions have found strong to moderate effect sizes in reducing depression, anxiety, and psychological distress and increasing well-being among participants compared with active controls [17]. Similarly, a systematic review of self-compassion interventions for workers indicated improvements not only in self-compassion but also in outcomes such as quality of life, well-being, and resilience as well as reduced stress and burnout [18]. Therefore, self-compassion is expected to improve workplace outcomes through mechanisms similar to those of mindfulness [13]. However, only a few randomized controlled trials (RCTs) have shown that self-compassion is effective in improving work-related outcomes; thus, a body of methodologically robust evidence is needed [18].

Both mindfulness and self-compassion training are centered on meditation practices and share the same emphasis on mindful states with acceptance, where events are experienced fully, as they are, without defensiveness [19]. Self-compassion further adds to the components of self-kindness and common humanity, which in turn contribute to mental health [20]. For low-intensity and short-period interventions, it is also important to select only effective content. Therefore, a comparative study of mindfulness and self-compassion practices, which are similar in terms of methods but may differ in effectiveness, is required.

### Challenges of Mindfulness and Self-Compassion

Traditional mindfulness and self-compassion programs are 8-week programs that include group sessions of approximately 2-3 hours each week, and participants are required to practice daily at home [8,21]. However, many employees have difficulty participating in formal programs due to strict work hours and the need to balance work and family life [22]. Web-based interventions can be a solution to the problem of supporting workers with time constraints. A growing body of research indicates that the use of digital technologies, such as smartphone apps and web-based platforms, may help overcome these barriers. For example, digitally assisted mindfulness interventions have been shown to effectively help participants develop a wide range of cognitive, emotional, and behavioral self-regulation skills, which has led to satisfying psychological needs and consequently enhancing mental wellness [23]. Mindfulness interventions that use state-of-the-art digital technology, such as virtual reality, an artificially created virtual space, are highly effective in overcoming barriers for people who have traditionally faced difficulties participating in mindfulness training [24]. According to Li et al [22], compared to traditional forms of intervention, web-based interventions are particularly suitable for employees. It has been shown that the essential skills of acceptance, mindfulness, and self-compassion can be acquired through low-intensity interventions delivered via smartphone apps [25]. Moreover, these app-based interventions have been found to reduce distress and job strain and improve workers’ well-being [5,26]. Although assistance with mobile health apps has been regarded as an effective tool for mental health support, little evidence of the effectiveness of smartphone app interventions in RCT designs exists, thus necessitating further research in rigorous designs [27]. Indeed, evidence is accumulating that self-care interventions using apps aimed at learning mindfulness and self-compassion principles reduce stress and improve attention and self-compassion [5]. When providing support to workers who are healthy enough to work, it is important to consider the effects on work-related outcomes and mental health. However, few studies have examined the impact of such app-based interventions on diverse work-related (eg, work performance) and mental health outcomes among workers.

The American Psychological Association has stated that the COVID-19 pandemic and the widespread adoption of remote work have affected the provision of psychological support for workers, and easy-to-access web-based psychological support is urgently required [28]. Thus, the need for remote web-based support, especially via highly convenient smartphone apps, has been recognized. Among other things, there is a need to develop a self-care app for workers to learn mindfulness and self-compassion meditation (SCM) methods and to evaluate the effectiveness of such an app using various indicators.

Virgili [29] has stated that a comprehensive understanding of the effectiveness of mindfulness-based interventions (MBIs) requires complementing self-reported psychological measures with objective physiological measures (eg, heart rate [HR] and HR variability [HRV]). HR and HRV are biomarkers indicating mental health status [30]. The normal regulation of HRV is dominated by parasympathetic activation; however, during stressful situations, sympathetic activation increases the HR. Low HRV can reflect autonomic nervous system inflexibility and has been considered a marker for unfavorable health outcomes [30]. It has been suggested that work stress might be associated with not only mental health deterioration but also increased HR and decreased HRV [31]. Although several studies have a high risk of bias, some reviews have shown that MBIs could reduce HR and increase HRV [32,33]. Furthermore, there is a need to examine the effects of MBI using objective and subjective indicators. Subjective indicators that request individuals to provide accounts of their experience depend on the individuals’ practice and experience in interrogating their minds, which is a skill that varies according to MBI [34]. However, most of the previous studies have focused on acute changes in HR and HRV effects during 1-session MBI or chronic changes measured before and after multiple-session MBI. Few studies have distinguished acute and chronic changes in multiple sessions [35]. This study uses a daily assessment approach. Daily intervention studies can be easily combined with a daily assessment approach to analyze within-person processes and individual differences in these processes [36]. Daily assessments have the following advantages: first, data are collected in real-world environments, as participants go about their lives [37]. HR and HRV may be particularly sensitive to circumstances. Second, the mechanism of intervention effects can be examined from daily fluctuations in physiological indicators [36]. Therefore, this study will examine the effects of the intervention from a physiological perspective by measuring HR and HRV, which can be measured non–face-to-face daily.

### Objectives

This study aims to develop a self-care app for mindfulness and SCM and examine its efficacy using mental health and work-related indicators. This study will use a 3-arm RCT design that divides the app-based intervention group into 2—the SCM group and the mindfulness meditation (MM) group—and includes a waitlist group. This study will adopt low-intensity intervention content to target busy workers who have had difficulty participating in traditional intervention forms. In addition, the intervention effects will be evaluated from a broad perspective by measuring mental health conditions and work-related outcomes such as work performance and physiological indicators. Thus, to gain insight into the mechanism of the effects, this study will examine effectiveness by not only collecting data on subjective measures but also measuring HR and HRV as objective physiological measures.

## Methods

### Participants

Recruitment began in December 2022 and ended in October 2023. Japanese-speaking participants were recruited from a broad Japanese worker community through an open call on Japanese websites, including a recruitment website for psychological experiments and our laboratory website in Japan. Contact information was provided in the recruitment guide, and participants could contact the research team directly.

The participants comprise young to middle-aged workers in Japan, working either full- or part-time. The inclusion criteria are employees who are (1) working more than 20 hours per week, (2) between the ages of 18 and 54 years, (3) not on a leave of absence, (4) not business owners or students, and (5) not currently diagnosed with a mental disorder and have a Kessler Psychological Distress Scale-6 (K6) score below 13 points. All these criteria will be assessed after informed consent is obtained, and participants who do not provide informed consent will be excluded. The smartphone app used in this study can only be used with an Apple iPhone (iOS); therefore, the participants are limited to iPhone users. The participants will not be informed that our study focuses on work performance or on the cutoff values for inclusion (ie, K6 score<13). Participants who meet the exclusion criteria according to their K6 cutoff scores will be informed by email that they are not eligible to participate. However, we will not provide specific reasons but only notify participants that they are not eligible to participate due to research reasons. In addition, participants who meet the exclusion criteria according to their K6 cutoff scores will receive an email with information about public psychological support services and health centers.

Interested candidates can read the experiment description posted on the website. Workers who are willing to participate can provide informed consent via the website. They will then be asked to confirm their consent to participate in this study and complete a screening questionnaire via a web form. The screening questionnaire consists of the K6 (screening for depression) and items regarding the inclusion criteria (participant attributes).

### Trial Design

This study will comprise an open-label randomized trial comparing an SCM course (n=67), an MM course (n=67), and a waitlist group (n=66). Based on the power analysis (see the *Sample Size* section), 37 participants are required in each active group (SCM and MM).

**Figure 1:**
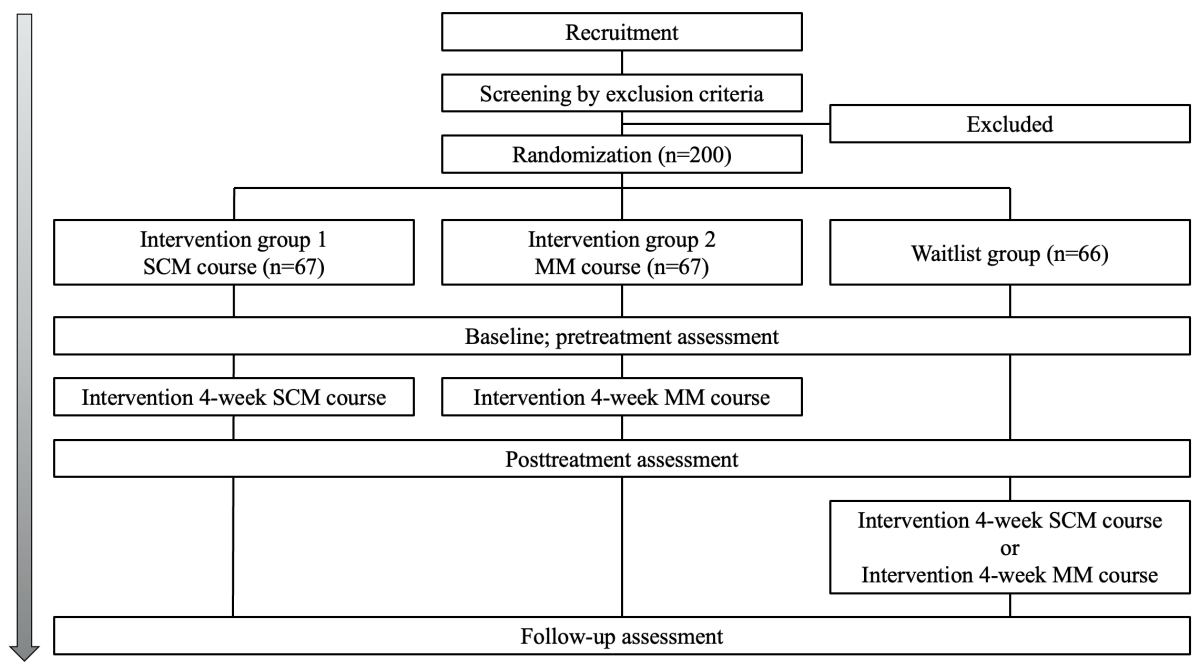
Intervention flowchart. MM: mindfulness meditation; SCM: self-compassion meditation.

The included participants will not be informed which arm they are allocated to but would be part of one of the intervention groups when the interventions commence. The waitlist group will be randomly assigned to either the SCM or MM group after a 1-month waiting period. Figure 1 presents the intervention flowchart.

### Interventions

SCM and MM were set as the intervention conditions and waitlist as the control condition, and the 3 groups were compared. Participants assigned to the SCM and MM groups will use a smartphone app to engage in guided meditation on their own once a day (Multimedia Appendix 1). The duration of all intervention conditions is 4 weeks. Both SCM and MM interventions contain elements of mindfulness. The difference between SCM and MM is the content of meditation in weeks 3 and 4: in SCM, participants will engage in loving-kindness meditation in weeks 3 and 4, whereas in MM, they will participate in a breath, sound, and body meditation. The duration of guided meditation is the same in both groups. Participants assigned to the waitlist group will receive no intervention during the 4-week waiting period; after the waiting period, they will be randomly assigned to either the SCM course or MM course to engage in 4 weeks of guided meditation.

The SCM and MM courses will be delivered via a smartphone app developed by the authors’ research team. Each course will offer 3 modules of guided self-help meditations. The modules in the SCM and MM courses will be structured as psychoeducation and guided meditation. The audio script for guided meditation is a Japanese version available from the University of California, Los Angeles Mindful Awareness Research Center [38], and audio instructions will be given before and after the meditation. The contents of psychoeducation include stress and stress responses and the theory of mindfulness and self-compassion. Both courses will involve the same guided meditations in weeks 1 and 2. The guided meditations contained in each week’s module are as follows (Table 1): week 1 (SCM and MM courses): a breathing meditation (7 minutes); week 2 (SCM and MM courses): a short body scan (6 minutes); and weeks 3-4: (SCM course): a loving-kindness meditation (12 minutes), including the contents of self-kindness and common humanity, and (MM course): a breath, sound, body meditation (12 minutes). Participants will receive weekly reminders via email to encourage involvement; however, there will be no minimum time commitment to participate.

**Table 1:** Schedule of procedures.

Process or assessment		Screening period	Pretreatment assessment (baseline)	Intervention period					Posttreatment assessment		Waitlist intervention period					Follow-up assessment
				Wk 1	Wk 2	Wk 3	Wk 4			Wk 1		Wk 2	Wk 3	Wk 4		
**Enrollment**																
	Eligibility screen	✓														
	Informed consent	✓														
	Allocation	✓														
**Assessment**																
	Demographic information		✓					✓							✓	
	K6^a^		✓					✓							✓	
	WHO-HPQ^b^		✓					✓							✓	
	SPS^c^		✓					✓							✓	
	SSS-8^d^		✓					✓							✓	
	CFS^e^		✓					✓							✓	
	RSES^f^		✓					✓							✓	
	SCS^g^		✓					✓							✓	
	PSS-14^h^		✓					✓							✓	
	SWLS^i^		✓					✓							✓	
	CERQ^j^		✓					✓							✓	
	UWES-9^k^		✓					✓							✓	
	STAXI-2^l^		✓					✓							✓	
	PSS^m^		✓					✓							✓	
	Creativity scale		✓					✓							✓	
**Weekly assessment**																
	Affect Grid			✓^n^	✓^n^	✓^n^	✓^n^			✓^o^		✓^o^	✓^o^	✓^o^		
	PANAS^p^			✓^n^	✓^n^	✓^n^	✓^n^			✓^o^		✓^o^	✓^o^	✓^o^		
**Daily assessment**																
	HR^q^ or HRV^r^			✓^n^	✓^n^	✓^n^	✓^n^			✓^o^		✓^o^	✓^o^	✓^o^		
	Relaxation scale			✓^n^	✓^n^	✓^n^	✓^n^			✓^o^		✓^o^	✓^o^	✓^o^		
**Interventions**																
	Breathing meditation			✓^n^						✓^o^						
	Short body scan				✓^n^							✓^o^				
	Loving-kindness meditation					✓^s^	✓^s^						✓^t^	✓^t^		
	Breath, sound, body meditation					✓^u^	✓^u^						✓^v^	✓^v^		

^a^K6: Kessler Psychological Distress Scale-6.

^b^WHO-HPQ: World Health Organization Health and Work Performance Questionnaire.

^c^SPS: Stanford Presenteeism Scale.

^d^SSS-8: Somatic Symptom Scale-8.

^e^CFS: Cognitive Flexibility Scale.

^f^RSES: Rosenberg Self-Esteem Scale.

^g^SCS: Self-Compassion Scale.

^h^PSS-14: Perceived Stress Scale-14.

^i^SWLS: Satisfaction With Life Scale.

^j^CERQ: Cognitive Emotion Regulation Questionnaire.

^k^UWES-9: Utrecht Work Engagement Scale-9.

^l^STAXI-2: State-Trait Anger Expression Inventory.

^m^PSS: Psychological Safety Scale.

^n^Self-compassion meditation and mindfulness meditation.

^o^Waitlist.

^p^PANAS: Positive and Negative Affect Schedule.

^q^HR: heart rate.

^r^HRV: heart rate variability.

^s^Self-compassion meditation.

^t^Waitlist self-compassion meditation.

^u^Mindfulness meditation.

^v^Waitlist mindfulness meditation.

### Criteria for Discontinuing or Modifying Allocated Interventions

In this study, only participants with psychological distress below a certain level will be included; thus, the risks associated with the intervention are likely to be minimal. However, we will provide information on support groups or mental health services that participants can contact in case they experience any distress. Participants can discontinue the trial at any time without providing further explanation.

### Strategies to Improve Adherence to Interventions

The smartphone app includes a reminder. Participants will receive a reminder once a day at a time of their own choice. Reminder notifications are not mandatory. Prior research has shown that reminders are a promising engagement strategy for encouraging adherence to web-based mindfulness programs [39].

### Provisions for Posttrial Care

All participants, including those assigned to the waitlist group, will attend a 4-week course in SCM or MM. Information on the mental health services operated by public institutions will be posted on a questionnaire. Participants will have access to mental health services as needed. If a participant makes an inquiry, we will introduce a similar application**.**

### Outcomes: Primary Outcome Measures

#### Psychological Distress

Psychological distress will be assessed using the Japanese version of K6 [40,41]. All items are rated on a scale ranging from 0=never to 4=always over the past 30 days. It has been designed to assess nonspecific psychological distress (depressive mood and anxiety) using 6 items. The reliability and validity of the Japanese version have been reported to be equal to those of the original English version [41]. A previous study suggested a cutoff point of 12 or 13 for K6 scores to screen for severe depressive symptoms [42]. Considering the possibility of a high psychological burden for participants with severe depressive symptoms, we set an exclusion criterion of a K6 cutoff value of 13 or higher, from an ethical point of view.

#### Work Performance

Presenteeism will be measured using the World Health Organization Health and Work Performance Questionnaire (WHO-HPQ) [43]. The Harvard Medical School stated that presenteeism is conceptualized as a measure of actual performance in relation to possible performance [44]. However, some studies have defined presenteeism as attending work while ill and have confirmed that it is associated with poor job performance [45]. This study follows the definition of Harvard Medical School, which developed the WHO-HPQ [44]. The WHO-HPQ provides 2 scores: the absolute presenteeism score, obtained by multiplying the self-reported single question to rate respondents’ overall work performance over the past 28 days by 10, and the relative presenteeism score, obtained by dividing the evaluation of others’ work by the self-reported single question score described earlier. In this study, absolute presenteeism scores will be used as an indicator of presenteeism. Absolute presenteeism scores range from 0=worst to 100=best. Additionally, regarding presenteeism scoring, the Harvard Medical School states that, unlike absenteeism, a higher score indicates a lower amount of lost performance [44]. Therefore, higher absolute presenteeism scores indicate higher work performance. Previous studies have confirmed the reliability and validity of the Japanese version of the scale [46,47]. Kessler et al [43] reported a close relationship between self-reported scores on the WHO-HPQ and the performance ratings by supervisors and peers.

Additionally, the work impairment score included in the Stanford Presenteeism Scale [48,49] will be used to assess work inefficiency. Work inefficiency is the state of one’s cognition, emotions, and behaviors that affect their ability to complete work and avoid distraction [50]. The subsequent studies follow this definition of work inefficiency by Turpin et al [50]. The work impairment score is measured using the responses given to the 10 Stanford Presenteeism Scale items on a 5-point scale where 1=always and 5=never. The validity and reliability of the Japanese version have been reported in a study on Japanese female workers [51].

### Outcomes: Secondary Outcome Measures

#### Somatic Symptom

The Somatic Symptom Scale-8 [52] can assess the somatic symptom burden during the previous 7 days. This scale includes 8 items rated on a 5-point Likert scale (0=not at all to 4=very much) with no reverse-coded items. The total score, ranging from 0 to 32, is the sum of all item scores, with a higher score indicating more severe somatic symptoms. The Japanese version of the Somatic Symptom Scale-8 has demonstrated acceptable reliability in a sample of Japanese individuals aged 20-64 years [53].

#### Cognitive Flexibility

The Cognitive Flexibility Scale (CFS) [54] is used to measure cognitive flexibility and considers an individual’s ability to produce diverse ideas, conceive response alternatives, and modify behaviors to adapt to changes in circumstances [55]. The CFS is a 12-item self-report questionnaire using a Likert-type scale ranging from 1=strongly disagree to 6=strongly agree. Each item in this questionnaire contains a sentence regarding beliefs and feelings about the respondent’s behavior. Total scores range from 12 to 72, with higher scores indicating greater cognitive flexibility. A previous study has confirmed the reliability and validity of the Japanese version of CFS among a sample of Japanese individuals [56].

#### Self-Esteem

Self-esteem will be measured using the Rosenberg Self-Esteem Scale [57], which consists of 10 items, such as “On the whole, I am satisfied with myself.” and “At times, I think I am not good at all.” Responses to these items are rated on a 4-point Likert scale (1=strongly disagree to 4=strongly agree). Mimura and Griffiths [58] have confirmed the reliability of the Japanese version.

#### Self-Compassion

The Self-Compassion Scale [16] is a 26-item assessment of the levels of self-compassion across 6 subscales (self-kindness, self-judgment, common humanity, isolation, mindfulness, and overidentification). Participants will be asked to rate the frequency of certain behaviors corresponding to each subscale. Responses range from 1=almost never to 5=almost always. The Self-Compassion Scale has good internal consistency, and its total score exhibits high convergent and discriminant validity [16]. The Japanese version of this inventory has high reliability and validity [59].

#### Perceived Stress

The Perceived Stress Scale-14 (PSS-14) is a self-report questionnaire intended to measure the degree of stress perceived by an individual in their life within the past month [60,61]. The PSS-14 consists of 14 items measuring how unpredictable, uncontrollable, and overloaded individuals feel about their circumstances. Participants rate each item on a 5-point Likert-type scale ranging from 0=never to 4=very often. Scores are obtained by reverse scoring the positively stated items and then summing the scores across all 14 items. The scores range from 0 to 56, with higher scores representing more significant perceived stress. Sumi [62] translated the Japanese version of the PSS-14 and confirmed its reliability and validity.

#### Well-Being

Life satisfaction will be assessed using the 5-item Satisfaction With Life Scale (SWLS) [63]. The SWLS items are rated on a 7-point Likert scale from 1=strongly disagree to 7=strongly agree, with higher scores indicating greater satisfaction with regard to one’s well-being. Its reliability and validity were substantiated by Pavot et al [64]. The Japanese version of the SWLS has been used in cross-cultural studies [65].

#### Emotion Regulation

The Cognitive Emotion Regulation Questionnaire is a 36-item scale measuring cognitive emotion regulation strategies to cope with the experience of threatening or stressful events or situations [66]. It evaluates 9 facets of cognitive handling strategies: positive reappraisal, putting into perspective, rumination or focus on thoughts, acceptance, self-blame, positive refocusing, other blame, catastrophizing, and refocus on planning. A 5-point Likert scale ranging from 1=almost never to 5=almost always is used to evaluate cognitive emotion regulation strategies. A validated Japanese version of the questionnaire will be used in this study [67].

#### Work Engagement

The Japanese version of the 9-item Utrecht Work Engagement Scale-9 can assess work engagement, which is defined as “a positive, fulfilling, work-related state of mind that is characterized by vigor, dedication, and absorption” [68-70]. Work engagement is assumed to be negatively correlated with burnout, and the 2 can be considered “opposites,” in that they represent a positive and a negative aspect of work-related well-being [70]. This scale includes measures of vigor, dedication, and absorption on a 7-point Likert scale from 0=never to 6=all the time. Each subscale comprises 3 items, and the scores of all are summed to determine the work engagement scores. The Utrecht Work Engagement Scale-9 has been translated into Japanese with adequate internal consistency and factorial validity [70].

#### Trait Anger

The State-Trait Anger Expression Inventory-2 is a 57-item questionnaire developed to measure the characteristic styles of coping with anger arousal using 5 components [71]. The 5 subscales include state anger, trait anger, anger-in, anger-out, and anger control. In this study, the participants will rate 10 items of the trait anger domain on a 4-point Likert scale ranging from 1=I strongly disagree to 4=I strongly agree to assess their degree of anger.

#### Psychological Safety

Psychological safety will be measured using the 7-item Psychological Safety Scale [72,73]. Psychological safety is defined as “being able to show and employ one’s self without fear of negative consequences to the self-image, status or career” [74]. Participants rate the items on a 7-point Likert-type scale ranging from 1=not at all to 7=strongly agree.

#### Creativity (Self-Perceived Creativity Scale)

The idea generation aspect of creativity in the workplace is assessed using 3 items from George and Zhou’s [75] scale modified for a self-report instrument by Dul et al [76]. This scale has been translated into Japanese and is used with the original authors’ permission. It includes the following 3 items for measuring employee creativity, which are scored on a 7-point Likert scale ranging from 1=do not agree to 7=agree: “In my work, I often have new and innovative ideas,” “In my work, I often come up with creative solutions to problems,” and “In my work, I often suggest new ways of performing work tasks.”

### Outcomes: Weekly Measures

#### Affect

The Affect Grid [77], which is used to assess emotional aspects, is a single-item scale designed as a quick means of assessing respondents’ affect along the dimensions of pleasure-displeasure and arousal-sleepiness. This scale evaluates the affect in a 2D structure consisting of 9×9 squares, with pleasure-displeasure on the horizontal axis and arousal-sleepiness on the vertical axis. The pleasure scores range from 1=unpleasurable to 9=highly pleasurable, and the arousal scores also range from 1=sleepy to 9=highly aroused.

#### Positive and Negative Emotions

The Positive and Negative Affect Schedule (PANAS) is used to measure the degree to which participants experienced positive and negative emotions within the past week [78]. The PANAS includes 20 items (10 positive and 10 negative emotions) that are rated on a 5-point Likert scale ranging from 1=not at all to 5=extremely. The Japanese version of this scale has demonstrated high reliability and validity [79].

### Outcomes: Daily Measures

Subjective psychological indices (relaxation) and physiological indices (HR and HRV) will be measured before and after daily meditation. The rating scale of relaxation will be used to assess the degree of participants’ relaxation [80]. This scale comprises 4 items, each of which is rated on a 10-point scale. Participants are required to “[select] the number from 1 to 10 that best describes [their] current state.” The 4 items are as follows: 1=feeling high to 10=stable, 1=tense to 10=relaxing, 1=anxiety to 10=relief, and 1=restrictive to 10=free. Nedate and Agari [80] used the average of the 4 scores as the relaxation score, which will be calculated similarly in this study as well (for more information on HR and HRV measurements, see the *Data Collection and Management* section).

### Participant Timeline

Participants who agree to participate in the study with informed consent and meet the inclusion criteria are assigned to the SCM, MM, or waitlist group. Participants are not notified as to which group they are assigned. All participants will then receive a web-based pretreatment assessment questionnaire at baseline via email and will be asked to complete it. Simultaneously, the participants assigned to the active arm (SCN and MM) will be asked via email to download and start using the smartphone app used for the intervention. Four weeks after the start of app use, participants will be sent a web-based posttreatment questionnaire by email and asked to complete it. Participants in the waitlist group will receive a web-based posttreatment questionnaire 4 weeks after they respond to the initial baseline questionnaire. Participants in the waitlist group will be asked to download and start using the smartphone app via email, distributed with the posttreatment questionnaire. All the participants in the waitlist group will be randomly assigned to the SCM and MM groups. During the 4-week intervention, all participants will be asked to respond to daily and weekly assessments of the smartphone app. Table 1 presents the schedule of procedures.

### Sample Size

The sample size is determined based on an a priori analysis of group-specific intervention effects. This analysis is modeled as the interaction of the within-subject factor of time (preintervention vs postintervention assessment) and the between-subject factor of the group (SCM vs MM vs control). Power analysis is conducted using the G*Power software app developed by Heinrich-Heine-Universität Düsseldorf Institut für Experimentelle Psychologie to estimate how much power would be required to find small interaction effects. In this study, the intervention effect size is assumed to have weak interaction effects (Cohen *F*=0.15) based on previous research [81]. Repeated measures scores are also expected to have a moderate to strong correlation of 0.5. To achieve 80% power, 37 participants are required in each arm (SCM, MM, and control). Expecting approximately 40% attrition based on similar previous studies [82,83], we will include 200 participants and assign 67 participants to each of the 3 arms.

### Recruitment

Recruitment will be conducted via a recruitment website for psychology experiments and through the website of our laboratory. Information about the study is available via posters on those websites. The posters also contain contact information for the research team, who can be contacted directly.

### Assignment of Interventions: Allocation

Randomization will be performed after obtaining informed consent and screening the participants. It will be computerized and independently carried out by research staff using a blocked randomization scheme (block size 15) to ensure the seamless participation of those who apply to participate in the study. Eligible participants will be randomized into the SCM, MM, or waitlist group in a ratio of 1:1:1.

Participants are assigned to either the intervention (SCM or MM) or control (waitlist) condition immediately after randomization. The intervention will begin approximately 1 week after allocation. No specific instructions are given to the waitlist group, and they will be asked to wait for 4 weeks.

Interested candidates will be asked to confirm their consent to participate in this study and complete a screening questionnaire via web forms. The screening questionnaire consists of the K6 and items based on the inclusion criteria. Participants who meet the inclusion criteria are immediately included in the blocked randomization scheme and assigned by the research staff. After the participants have been assigned, they will receive an email with instructions to participate or wait.

### Assignment of Interventions: Blinding

It is practically impossible to blind the allocation in this study because the participants engage in the meditation independently, following a guide. Therefore, this study will be an open-label RCT. Once a participant is assigned to a condition, there will be no further interaction with the research staff, except when the participant contacts the research staff. To minimize interaction between the participants and research staff, notifications and instructions will be sent to the participants via automated emails. Participants can infer which group they have been allocated to, based on the instructions, but they are not explicitly informed about the groups to which they are assigned.

### Data Collection and Management

The participants will be assessed for outcome measures via web forms at baseline (pretreatment assessment) and 1 month and 2 months after the course starts (posttreatment assessment and follow-up assessment, respectively). Additionally, the participants will complete weekly and daily assessments during their courses. As mentioned earlier, the weekly measures consist of 1 question from the Affect Grid [77] and 20 questions from the PANAS [78,79]. The daily assessment involves the degree of participants’ relaxation [80] and objective physiological measures (HR and HRV) on the smartphone app. Participants will be asked to perform daily assessments using the smartphone app before and after daily meditation. The weekly assessment will be measured via web-based forms once a week immediately after the 7-day meditations, and participants will receive an email requesting them to respond to the questionnaire and a reminder to use the self-care app. The web-based survey to be conducted in this study is designed with reference to the CHERRIES (Checklist for Reporting Results of Internet E-Surveys) checklist to ensure a complete description of web-based surveys (Multimedia Appendix 2) [84]. When reporting the results, we will provide a detailed report of what is described in CHERRIES.

HR and HRV measurements will be taken on the smartphone app used for the intervention. Participants will be provided a sample and instructed to gently press their index finger against their smartphone’s camera for 30 seconds. At this time, the smartphone’s flash will turn on. By capturing the finger illuminated by the flash with the smartphone camera, HR and HRV will be measured. The procedure was set up based on a study that used HR and HRV measurements to evaluate the effectiveness of psychological interventions similar to this study [81]. HR and HRV will be measured using contact photoplethysmography, which is a noninvasive and convenient optical biomonitoring technique used to detect pulsatile changes in blood volume that appear in the microvascular bed of tissues [85]. The contact photoplethysmography method uses a white LED flashlight built into a smartphone and a rear camera to illuminate the skin for 30 seconds and detect light absorption due to arterial pulsation in the finger pad (Multimedia Appendix 3). For measuring HRV, the root mean square of successive differences—a measure of the square root of the mean squared difference between consecutive normal heartbeats—is used. The average HR measured in beats per minute is analyzed for HR.

When participants do not answer a questionnaire (baseline, preintervention assessment, and postintervention assessment), they will receive automatic reminders via email 6 days later. Participants who do not respond at baseline and preintervention assessment will receive further reminders after 13 days. If the participants are found not to respond to the questionnaire, they will be considered dropped by 14 days at baseline and preintervention assessment and by 7 days at postintervention assessment.

The data collected through the web-based forms are stored on a secure server with restricted access at the University of Tokyo. After data collection, this study will use a study-generated identification for data management, analysis, and publication. The list linking identifications to the participants’ personal information is stored on a hard disk disconnected from the internet, and the hard disk is kept in a locked cabinet.

This study will be conducted according to the protocol approved by the ethics committee of the institution to which the research team belongs. Identifications are used only for data management, and collected data and personal information are stored separately. Participants are informed that their personal information will remain confidential if they provide informed consent.

### Statistical Methods

#### Primary Outcome

The intention-to-treat approach involving participants who have completed pretreatment and posttreatment assessments will be used to test the effectiveness of the active arms compared to the waitlist group. To examine preintervention and postintervention changes and pre-post differences between the groups, the results of the assessment questionnaires will be analyzed using repeated measures ANOVA. The statistical significance level is set at *P*<.05 for all statistics. Analyses will be performed using SPSS (IBM Corp) and R (R Foundation for Statistical Computing).

#### Secondary Outcomes

Descriptive statistics of the study participant characteristics at baseline and questionnaire responses will include means, SDs, frequencies, and percentages according to the type of variable (continuous or categorical). The fit of quantitative variables to the normal distribution will be confirmed by the Shapiro-Wilk test. Differences in demographic information at baseline between groups will be analyzed using the chi-square test for categorical variables and ANOVA or the 2-tailed *t* test for continuous variables (ie, age, sex, employment status, educational attainment, previous meditation experience, and psychological distress at baseline).

#### Other Analyses

An interim analysis will be conducted once half of the planned number of participants have completed the intervention period. The interim analysis will primarily examine the feasibility, including dropout rates, and the trial may be discontinued if the dropout rate is higher than expected. Multivariate analysis will be conducted to test the effects of the interventions, adjusting for estimated predictors or confounding variables, and multilevel linear regression will be performed.

Following the intention-to-treat principle, the analyses will include data from all participants. Web-based self-care interventions are often associated with higher dropout rates and lower rates of continued implementation [86]. In their study, Farvolden et al [87] reported that only 12 of 1161 participants (approximately 1%) completed a 12-week web-based panic disorder self-help program in its entirety. Similarly, Hasselberg and Rönnlund [83] conducted a 2-week web-based program targeting mindfulness and self-compassion and reported that 33% of the participants stayed in the program until the end. Therefore, even if self-care app activities are minimal, participants will be considered to have completed the course and will be included in the analysis if each questionnaire is answered. The patterns of missing values will be examined and, when applicable, replaced with suitable statistical methods.

### Adverse Event Reporting and Harms

The participants in this study comprise a nonclinical population with lower than a certain level of psychological distress and no diagnosed mental disorders. Therefore, the risk associated with psychological interventions is assumed to be low. Participants may interrupt a trial without disclosing the reason and will be able to contact the research staff via email or web-based forms, if necessary. Participants can also obtain information on mental health services operated by public institutions on web-based forms and receive mental health care as needed.

### Dissemination Plans

The findings of this study will be disseminated through presentations at national and international conferences and publications in international journals. The results of this research will adhere to the CONSORT (Consolidated Standards of Reporting Trials) statement [88].

### Ethical Considerations

The study protocol used in this study and all procedures were approved by the Life Science Research Ethics and Safety Committee of the University of Tokyo (22-326). Data collected in this study will be anonymized and managed. Written informed consent to participate will be obtained from all participants via a web form. Participants are given the option to opt out at any time. Participants will also be rewarded with a gift card worth up to JP ¥10,000 (JP ¥137.09=US $1 as of December 1, 2022), depending on their level of participation.

## Results

Recruitment of participants began in December 2022, and the intervention began in January 2023. A total of 375 participants have been enrolled as of September 2023. The intervention and data collection will be completed by late October 2023.

## Discussion

### Principal Findings

The purpose of this study is to develop a self-care app for MM and SCM and examine its effectiveness using various indicators such as work performance. The study will adopt an RCT design comparing the SCM and MM intervention groups and a waitlist group. This study will focus on accessibility and availability and will use a low-intensity self-care program delivered via a smartphone app. This will allow us to examine the possibility of supporting busy workers who have had difficulty participating in traditional intervention formats. It is expected that by developing accessible self-care smartphone apps and examining the effectiveness of the intervention, it will be possible to propose efficient and feasible solutions for improving workers’ mental health and work performance. In the field of worker assistance, where methodologically robust evidence is scarce [18], this study can contribute to the body of findings by examining the effectiveness of assistance through an RCT design.

In recent years, health support technologies using mobile digital devices, such as smartphones, have been developed, and their effectiveness has been examined; however, comparability between studies has been considered difficult [5]. Using consistent guidelines—such as the mobile health evidence reporting and assessment checklist [89]—when reporting the results of this study ensure that the findings from this study provide synthesizable evidence and promote comparability between studies.

### Strengths and Limitations

The study has several notable strengths. First, this study will adopt brief self-care interventions for preventive health care in healthy workers. Traditional self-compassion and mindfulness interventions are long-term face-to-face programs [8,21], thus presenting difficulties related to worker participation and dissemination [22]. For interventions aimed at treatment, evidence-based, structured programs for well-experienced instructors are essential. However, even a brief intervention with an app may be sufficient to facilitate preventive health care for healthy workers.

Second, this study will examine the intervention effects not only on mental health conditions but also on work-related outcomes and objective physiological measures, such as HR and HRV. Examining work-related outcomes can provide a broad perspective of the effects of interventions. Measuring objective physiological outcomes may provide insights into the mechanisms underlying these effects.

Third, this RCT design compares 2 types of interventions in the intervention group: SCM and MM. They are similar but have different background theories. This study on preventive health care for healthy workers has the potential to provide suggestive findings for the development of accessible, available, and more effective intervention content that can improve mental health and work-related outcomes.

This study has several limitations. First, the duration of the intervention is shorter, and the intensity is lower than that of traditional self-compassion and mindfulness interventions. Second, the intervention is conducted in a self-care format, which may result in a lack of sufficient skill acquisition and limited intervention effectiveness. Third, because the target population is limited to healthy workers, the intervention effect may be too small and not be fully confirmed. Furthermore, a trait mindfulness scale is not included in this study’s secondary outcomes. Measuring both, a self-compassion measure and a trait mindfulness scale would have provided a better understanding of each intervention’s effects and allowed us to determine any differences in the constructs being targeted. However, this is not possible in this study. Therefore, future research should use both a self-compassion measure and a trait mindfulness scale to better understand the intervention effects.

### Conclusions

The intervention protocol in this study has described an RCT to examine whether brief guided MM or SCM interventions provided by a smartphone app are effective for mental health and work-related outcomes among workers. The authors’ research team at the University of Tokyo, Japan, planned to begin the RCT in December 2022. There is a great need for preventive interventions for mental health and work-related problems such as presenteeism in the working population because of inadequate support. We expect that this study will contribute to the evaluation of effective self-care intervention content that will improve mental health, work performance, and related outcomes and promote mindful and self-compassionate attitudes when faced with distress.

## Appendix

Multimedia Appendix 1 App screenshot 1.

Multimedia Appendix 2 The CHERRIES (Checklist for Reporting Results of Internet E-Surveys) checklist.

Multimedia Appendix 3 App screenshot 2.

## Abbreviations

CFS: Cognitive Flexibility Scale
CHERRIES: Checklist for Reporting Results of Internet E-Surveys
CONSORT: Consolidated Standards of Reporting Trials
HR: heart rate
HRV: heart rate variability
K6: Kessler Psychological Distress Scale-6
MBI: mindfulness-based intervention
MM: mindfulness meditation
PANAS: Positive and Negative Affect Schedule
PSS-14: Perceived Stress Scale-14
RCT: randomized controlled trial
SCM: self-compassion meditation
SWLS: Satisfaction With Life Scale
WHO-HPQ: World Health Organization Health and Work Performance Questionnaire

## Data Availability

The data sets generated and analyzed during this study are not publicly available because data sharing in publicly available repositories was not included in the informed consent. However, the data sets generated and analyzed during this study are available from the corresponding author on reasonable request.
