# Supplementary figures and images for: Mindful Self-Compassion Smartphone Intervention for Worker Mental Health in Japan: Protocol for a Randomized Controlled Trial
Source: JMIR Res Protoc. 2024 Jul 15;13:e53541. doi: 10.2196/53541 (PMC11287101; doi:10.2196/53541)

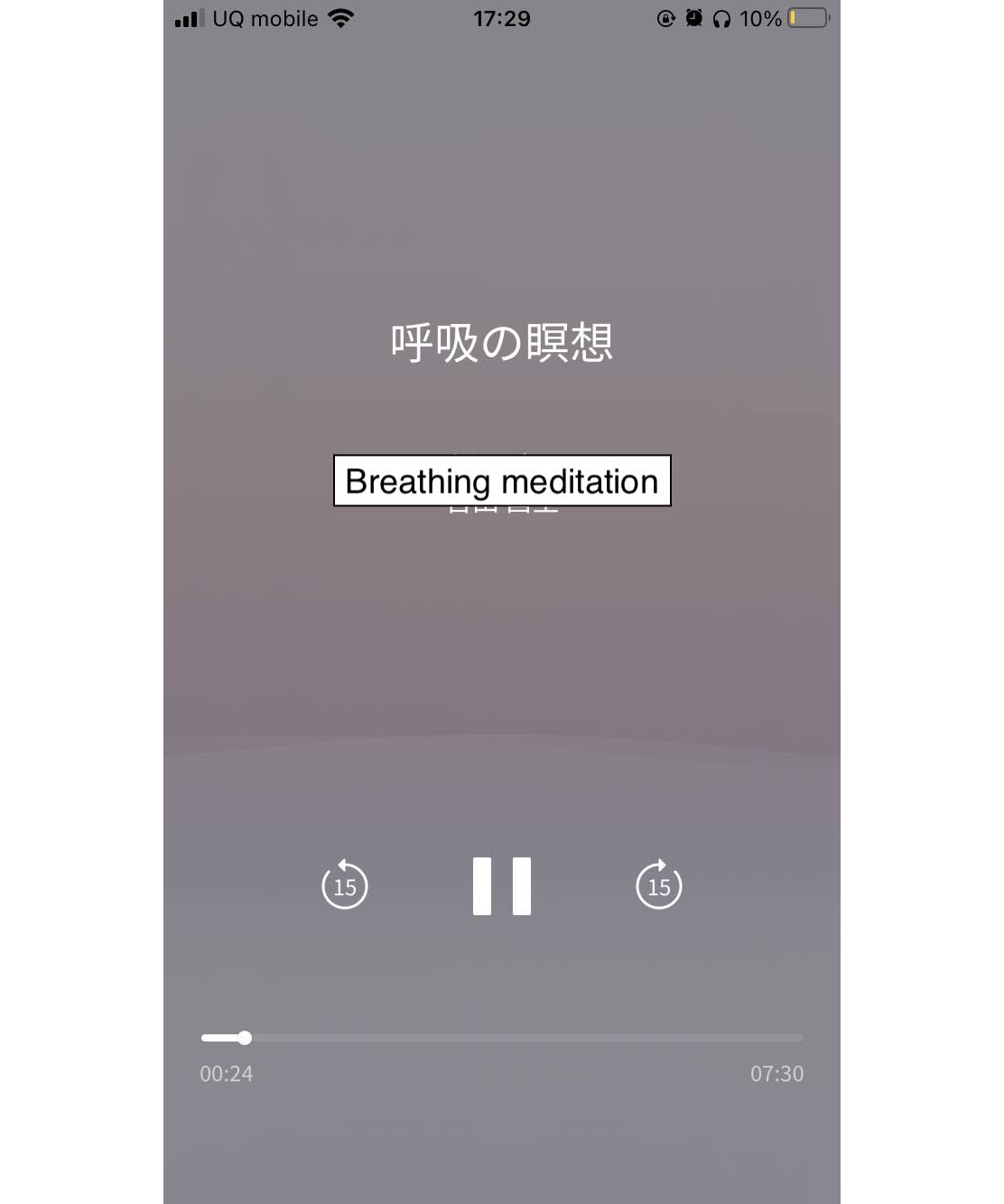

Supplement: Multimedia Appendix 1 [file resprot_v13i1e53541_app1.png]

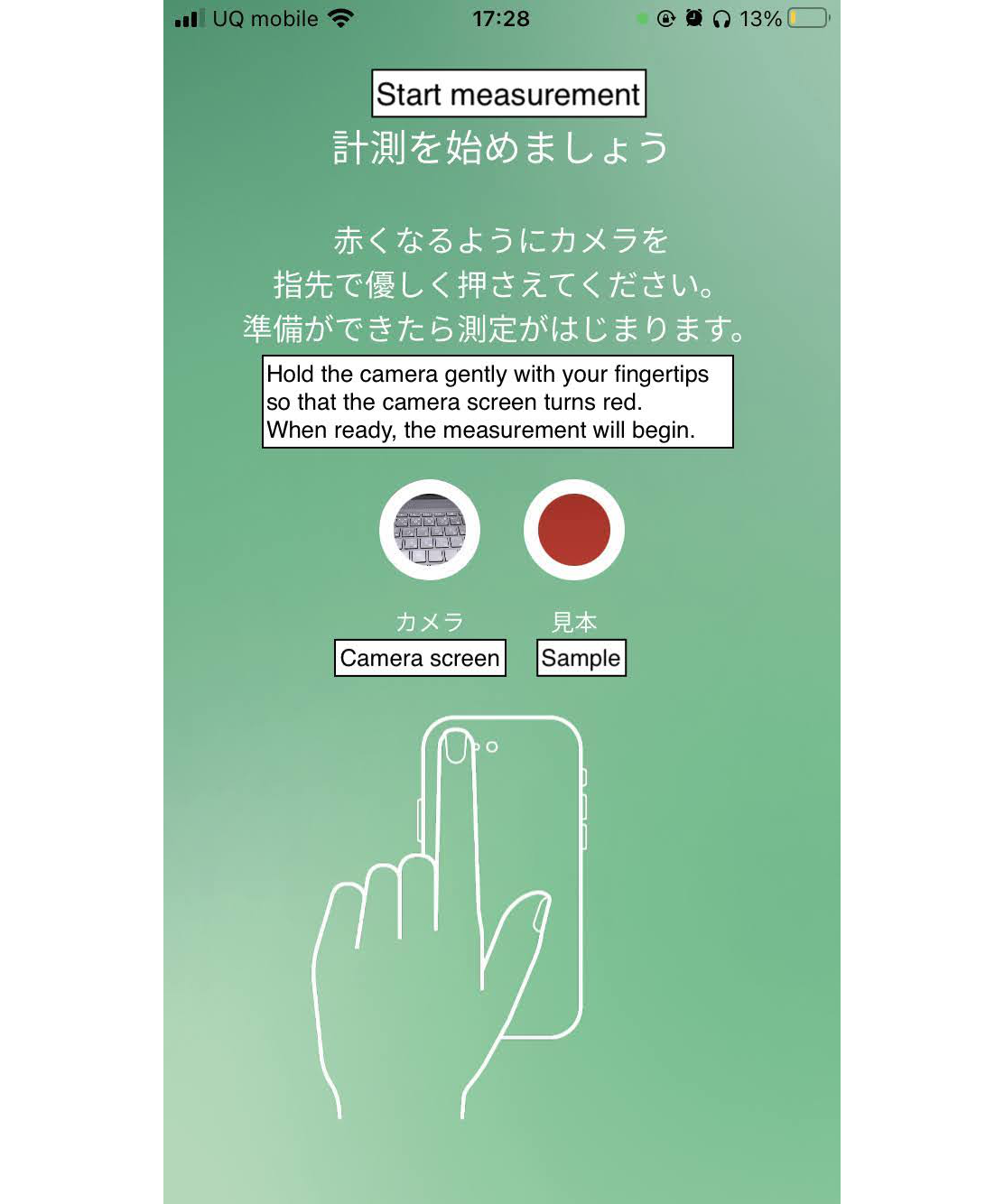

Supplement: Multimedia Appendix 3 [file resprot_v13i1e53541_app3.png]
