# Supplementary material for: Mindful Self-Compassion Smartphone Intervention for Worker Mental Health in Japan: Protocol for a Randomized Controlled Trial
Source: JMIR Res Protoc. 2024 Jul 15;13:e53541. doi: 10.2196/53541 (PMC11287101; doi:10.2196/53541)
Supplement: Multimedia Appendix 2 [file resprot_v13i1e53541_app2.docx]

**Checklist for Reporting Results of Internet E-Surveys (CHERRIES)**

| ***Checklist Item*** | ***Explanation*** | ***Evaluation in this study*** |
| --- | --- | --- |
| Describe survey design | Describe target population, sample frame. Is the sample a convenience sample? (In “open” surveys this is most likely.) | Target population: (1) Employees who are working more than 20 h per week; (2) between the ages of 18 and 54 years; (3) not on a leave of absence; (4) not business owners or students; and (5) not currently diagnosed with a mental disorder, and have a Kessler Psychological Distress Scale (K6) score of less than 13 points.  Sample: The above must be satisfied, and iPhone users who have viewed the call for research announcement. |
| IRB approval | Mention whether the study has been approved by an IRB. | The study protocol used in this study and all procedures were approved by the Life Science Research Ethics and Safety Committee of the University of Tokyo (No. 22-326). |
| Informed consent | Describe the informed consent process. Where were the participants told the length of time of the survey, which data were stored and where and for how long, who the investigator was, and the purpose of the study? | Workers who are willing to participate can provide informed consent via the website. Participants will find out the time required on the website. The data collected through the online forms are stored on a secure server with restricted access at the University of Tokyo. The survey will be conducted by K.T. and A.K. The purpose of the study is to develop a self-care app for mindfulness and SCM and to examine its efficacy using mental health and work-related indicators. |
| Data protection | If any personal information was collected or stored, describe what mechanisms were used to protect unauthorized access. | This study will use a study-generated identification (ID) for data management, analysis, and publication. The list linking IDs to the participants' personal information is stored on a hard disk disconnected from the internet and the hard disk is kept in a locked cabinet. |
| Development and testing | State how the survey was developed, including whether the usability and technical functionality of the electronic questionnaire had been tested before fielding the questionnaire. | First, create a web form to be used for the survey. Before the survey begins, we will ask members of the research team to test the usability and technical functionality of the electronic questionnaire. They will also be asked to respond to the time required. |
| Open survey versus closed survey | An “open survey” is a survey open for each visitor of a site, while a closed survey is only open to a sample which the investigator knows (password-protected survey). | The registration form is open to all visitors to the site. The survey form is open only to participants who meet the inclusion criteria and participate in the study. |
| Contact mode | Indicate whether or not the initial contact with the potential participants was made on the Internet. (Investigators may also send out questionnaires by mail and allow for Web-based data entry.) | Initial contact is made on the Internet. |
| Advertising the survey | How/where was the survey announced or advertised? Some examples are offline media (newspapers), or online (mailing lists – If yes, which ones?) or banner ads (Where were these banner ads posted and what did they look like?). It is important to know the wording of the announcement as it will heavily influence who chooses to participate. Ideally the survey announcement should be published as an appendix. | Recruitment will be conducted via a recruitment website for psychology experiments and through the website of our laboratory. Information about the study is available via posters on those websites. |
| Web/E-mail | State the type of e-survey (eg, one posted on a Web site, or one sent out through e-mail). If it is an e-mail survey, were the responses entered manually into a database, or was there an automatic method for capturing responses? | Participants will be provided with the URL of the web form via email. All surveys will be conducted on the web form and responses will be automatically captured and saved. |
| Context | Describe the Web site (for mailing list/newsgroup) in which the survey was posted. What is the Web site about, who is visiting it, what are visitors normally looking for? Discuss to what degree the content of the Web site could pre-select the sample or influence the results. For example, a survey about vaccination on a anti-immunization Web site will have different results from a Web survey conducted on a government Web site | The following are the sites where the recruitment will take place.  The recruitment website for psychology experiments: The site is designed to recruit participants for research and experiments in psychology and other fields. People browsing this site are seeking information to participate or cooperate in research. It is expected that some participants will have participated in other studies before.  The website of our laboratory: This is the home page of the laboratory. People interested in the research team will visit. People who visit this homepage are likely to have a deep knowledge of psychology and research. |
| Mandatory/voluntary | Was it a mandatory survey to be filled in by every visitor who wanted to enter the Web site, or was it a voluntary survey? | This survey is voluntary. |
| Incentives | Were any incentives offered (eg, monetary, prizes, or non-monetary incentives such as an offer to provide the survey results)? | Rewards are offered as monetary (¥7,000-10,000 for completing the experiment). The breakdown is ¥3,000 for conducting the meditation on the app and ¥1,000 7 times for each survey response. |
| Time/Date | In what timeframe were the data collected? | The participants will be assessed for outcome measures via web forms at baseline (pretreatment assessment) and 1 month and 2 months after the course starts (post-treatment assessment and follow-up assessment, respectively). Additionally, the participants will complete weekly and daily assessments during their courses. Participants will be asked to perform daily assessments using the smartphone app before and after daily meditation. The weekly assessment will be measured via online forms once a week immediately after the seven days’ meditations. |
| Randomization of items or questionnaires | To prevent biases items can be randomized or alternated. | No randomization of items or questionnaires. |
| Adaptive questioning | Use adaptive questioning (certain items, or only conditionally displayed based on responses to other items) to reduce number and complexity of the questions. | Adaptive questioning methods are not used. |
| Number of Items | What was the number of questionnaire items per page? The number of items is an important factor for the completion rate. | Application form questionnaires: P.1 2 items, P.2 6 items  Baseline questionnaires: P.1 9 items, P.2 7 items, P.3 13 items, P.4 2 items, P.5 3 items, P.6 12 items, P.7 9 items, P.8 10 items, P.9 39 items, P.10 4 items, P.11 10 items  Pre-, post- and follow-up intervention questionnaires: P.1 6 items, P.2 8 items, P.3 12 items, P.4 10 items, P.5 26 items, P.6 14 items, P.7 4 items, P.8 36 items, P.9 3 items, P.10 3 items, P.11 9 items, P.12 10 items, P.13 7 items, P.14 3 items, P.15 10 items  Weekly questionnaires: P.1 1 item, P.2 1 item, P.3 20 items  Daily questionnaires: P.1 1 item, P.2 1 item, P.3 1 item, P.4 1 item |
| Number of screens (pages) | Over how many pages was the questionnaire distributed? The number of items is an important factor for the completion rate. | Application form questionnaires: 2 pages  Baseline questionnaires: 11pages  Pre-, post- and follow-up intervention questionnaires: 15pages  Weekly questionnaires: 3 pages  Daily questionnaires: 4 pages |
| Completeness check | It is technically possible to do consistency or completeness checks before the questionnaire is submitted. Was this done, and if “yes”, how (usually JAVAScript)? An alternative is to check for completeness after the questionnaire has been submitted (and highlight mandatory items). If this has been done, it should be reported. All items should provide a non-response option such as “not applicable” or “rather not say”, and selection of one response option should be enforced. | Consistency and completeness will not be checked prior to submittal. Responses to the questions are not required. |
| Review step | State whether respondents were able to review and change their answers (eg, through a Back button or a Review step which displays a summary of the responses and asks the respondents if they are correct). | Respondents can review and change their answers using the back button. A summary of the responses will not be displayed. |
| Unique site visitor | If you provide view rates or participation rates, you need to define how you determined a unique visitor. There are different techniques available, based on IP addresses or cookies or both. | N/A. It is a protocol and does not present. |
| View rate (Ratio of unique survey visitors/unique site visitors) | Requires counting unique visitors to the first page of the survey, divided by the number of unique site visitors (not page views!). It is not unusual to have view rates of less than 0.1 % if the survey is voluntary. | N/A. It is a protocol and does not present. |
| Participation rate (Ratio of unique visitors who agreed to participate/unique first survey page visitors) | Count the unique number of people who filled in the first survey page (or agreed to participate, for example by checking a checkbox), divided by visitors who visit the first page of the survey (or the informed consents page, if present). This can also be called “recruitment” rate. | N/A. It is a protocol and does not present. |
| Completion rate (Ratio of users who finished the survey/users who agreed to participate) | The number of people submitting the last questionnaire page, divided by the number of people who agreed to participate (or submitted the first survey page). This is only relevant if there is a separate “informed consent” page or if the survey goes over several pages. This is a measure for attrition. Note that “completion” can involve leaving questionnaire items blank. This is not a measure for how completely questionnaires were filled in. (If you need a measure for this, use the word “completeness rate”.) | N/A. It will be calculated by dividing the number of people who submitted the last survey page by the number of people who agreed to participate, but will not be reported at this time due to the protocol paper. |
| Cookies used | Indicate whether cookies were used to assign a unique user identifier to each client computer. If so, mention the page on which the cookie was set and read, and how long the cookie was valid. Were duplicate entries avoided by preventing users access to the survey twice; or were duplicate database entries having the same user ID eliminated before analysis? In the latter case, which entries were kept for analysis (eg, the first entry or the most recent)? | Cookies are not used. |
| IP check | Indicate whether the IP address of the client computer was used to identify potential duplicate entries from the same user. If so, mention the period of time for which no two entries from the same IP address were allowed (eg, 24 hours). Were duplicate entries avoided by preventing users with the same IP address access to the survey twice; or were duplicate database entries having the same IP address within a given period of time eliminated before analysis? If the latter, which entries were kept for analysis (eg, the first entry or the most recent)? | To avoid duplicate entries from the same user, use a web form that requires a login. However, IP addresses are not used. |
| Log file analysis | Indicate whether other techniques to analyze the log file for identification of multiple entries were used. If so, please describe. | To avoid multiple entries, use a web form that requires a login. However, log file analysis techniques are not used to multiple entries. |
| Registration | In “closed” (non-open) surveys, users need to login first and it is easier to prevent duplicate entries from the same user. Describe how this was done. For example, was the survey never displayed a second time once the user had filled it in, or was the username stored together with the survey results and later eliminated? If the latter, which entries were kept for analysis (eg, the first entry or the most recent)? | Participants who register on the application form and meet the inclusion criteria are assigned a unique ID and asked to enter the ID when they respond. The first entry is used in the analysis. |
| Handling of incomplete questionnaires | Were only completed questionnaires analyzed? Were questionnaires which terminated early (where, for example, users did not go through all questionnaire pages) also analyzed? | Even for incomplete questionnaires, the items answered will be analyzed. |
| Questionnaires submitted with an atypical timestamp | Some investigators may measure the time people needed to fill in a questionnaire and exclude questionnaires that were submitted too soon. Specify the timeframe that was used as a cut-off point, and describe how this point was determined. | The response start time cannot be recorded on the web form. Therefore, the time taken to answer the questionnaire cannot be measured and the questionnaire is not deleted by time stamp. |
| Statistical correction | Indicate whether any methods such as weighting of items or propensity scores have been used to adjust for the non-representative sample; if so, please describe the methods. | No statistical correction is made. |

This checklist has been modified from Eysenbach G. Improving the quality of Web surveys: the Checklist for Reporting Results of Internet E-Surveys (CHERRIES). J Med Internet Res. 2004 Sep 29;6(3):e34 [erratum in J Med Internet Res. 2012; 14(1): e8.]. Article available at [https://www.jmir.org/2004/3/e34](https://www.jmir.org/2004/3/e34/)/; erratum available <https://www.jmir.org/2012/1/e8/>. Copyright ©Gunther Eysenbach. Originally published in the [Journal of Medical Internet](http://www.jmir.org) Research, 29.9.2004 and 04.01.2012.

This is an open-access article distributed under the terms of the Creative Commons Attribution License (<https://creativecommons.org/licenses/by/2.0/>), which permits unrestricted use, distribution, and reproduction in any medium, provided the original work, first published in the Journal of Medical Internet Research, is properly cited.
